# Frequency and bandwidth conversion of single photons in a room-temperature diamond quantum memory

**DOI:** 10.1038/ncomms11200

**Published:** 2016-04-05

**Authors:** Kent A. G. Fisher, Duncan G. England, Jean-Philippe W. MacLean, Philip J. Bustard, Kevin J. Resch, Benjamin J. Sussman

**Affiliations:** 1 Institute for Quantum Computing and Department of Physics and Astronomy, University of Waterloo, 200 University Avenue West, Waterloo, Ontario, Canada N2L 3G1; 2 National Research Council of Canada, 100 Sussex Drive, Ottawa, Ontario, Canada K1A 0R6; 3 Department of Physics, University of Ottawa, 150 Louis Pasteur, Ottawa, Ontario, Canada K1N 6N5

## Abstract

The spectral manipulation of photons is essential for linking components in a quantum network. Large frequency shifts are needed for conversion between optical and telecommunication frequencies, while smaller shifts are useful for frequency-multiplexing quantum systems, in the same way that wavelength division multiplexing is used in classical communications. Here we demonstrate frequency and bandwidth conversion of single photons in a room-temperature diamond quantum memory. Heralded 723.5 nm photons, with 4.1 nm bandwidth, are stored as optical phonons in the diamond via a Raman transition. Upon retrieval from the diamond memory, the spectral shape of the photons is determined by a tunable read pulse through the reverse Raman transition. We report central frequency tunability over 4.2 times the input bandwidth, and bandwidth modulation between 0.5 and 1.9 times the input bandwidth. Our results demonstrate the potential for diamond, and Raman memories in general, as an integrated platform for photon storage and spectral conversion.

## Abstract (web summary)

Controlling the spectral properties of single photons is important for emerging optical quantum technologies, but doing so in a frequency-multiplexed framework is challenging. Here, the authors demonstrate quantum frequency conversion with a Raman quantum memory in room-temperature diamond.

## Graphical abstract

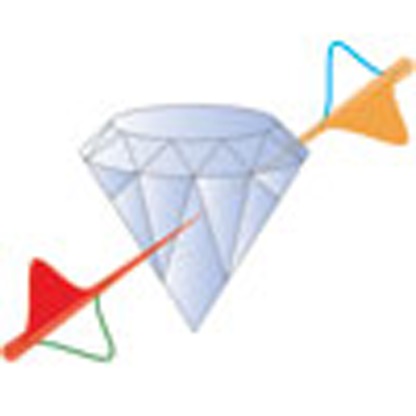

The fragility of the quantum state is a challenge facing all quantum technologies. Great efforts have been undertaken to mitigate the deleterious effects of decoherence by isolating quantum systems, for example, by cryogenically cooling and isolating in vacuum. State-of-the-art decoherence times are now measured in hours^1^. An alternative approach is to build quantum technologies that execute on ultrafast timescales—as short as femtoseconds—such that operations can be completed before decoherence overwhelms unitarity. A shining example is the Raman quantum memory^2^,^3^,^4^,^5^, which can absorb single photons of femtosecond duration and release them on demand several picoseconds later^6^. While picosecond storage times are not appropriate for conventional quantum memory applications such as long-distance communication, it has been suggested^2^ that Raman quantum memories can find additional uses such as frequency and bandwidth conversion.

Controlling the spectral properties of single photons is essential for a wide array of emerging optical quantum technologies spanning quantum sensing^7^, quantum computing^8^ and quantum communications^9^. Essential components for these technologies include single-photon sources^10^, quantum memories^11^, waveguides^12^ and detectors^13^. The ideal spectral operating parameters (wavelength and bandwidth) of these components are rarely similar; thus, frequency conversion and spectral control are key enabling steps for component hybridization^14^. Beyond hybridization, frequency conversion is an area of emerging interest in quantum optical processing. The frequency degree of freedom can be used along side conventional encoding in, for example, polarization, or time-bin, to build quantum states of higher dimensionality^15^.

Spectral control is a mature field in ultrafast optics where phase- and amplitude-shaping of a THz-bandwidth pulse can be achieved using passive pulse-shaping elements in the Fourier plane^16^. Meanwhile, a range of nonlinear optical techniques^17^ such as second harmonic generation (SHG), sum- and difference-frequency generation, four-wave mixing and Raman scattering are routinely employed to shift the frequency of laser pulses. Extending these frequency conversion techniques into the quantum regime is a critical task for many quantum technologies but is made difficult by the low intensity of single photons, and the sensitivity of quantum states to loss and noise. Despite these challenges, quantum frequency conversion^18^ has been demonstrated in a number of systems including waveguides in nonlinear crystals^19^,^20^,^21^,^22^,^23^,^24^, photonic crystal fibres^25^ and atomic vapour^26^. Similarly, photon bandwidth compression has been shown using chirped-pulse upconversion^27^. Full control over the spectral properties of single photons^28^ has been proposed using second-14,29 and third-order^30^ optical nonlinearities.

Large frequency shifts, such as those achieved using sum- and difference-frequency generation, are desirable to convert photons to, and from, the telecommunication band. Meanwhile, smaller shifts can be useful for frequency-multiplexing in several closely spaced bins. This concept is widely used in classical fibre optics, where wavelength division multiplexing is employed to achieve data rates far beyond that which could be achieved with monochromatic light^31^. However, in quantum optics the utility of frequency multiplexing has only recently been explored^15^,^32^,^33^,^34^. In a frequency-multiplexed quantum architecture, it will be critical to build components that can add, drop, and manipulate different frequency bins: it has been proposed that frequency-selective quantum memories can perform this task^15^,^33^. As with classical wavelength division multiplexing, the frequency bins will likely be closely spaced so that small shifts around a central frequency will be required.

In this article, we demonstrate the use of a Raman quantum memory to perform quantum frequency conversion; we manipulate the spectral properties of THz-bandwidth photons using a memory in the optical phonon modes of diamond^6^. Crucially, the quantum properties of the photon must be maintained even while the carrier frequency and bandwidth are modified. In our demonstration, a signal photon is mapped into an optical phonon by the write pulse, and then retrieved with its spectral properties modified according to the properties of the read pulse.

## Results

### Experiment

**Figure 1:**
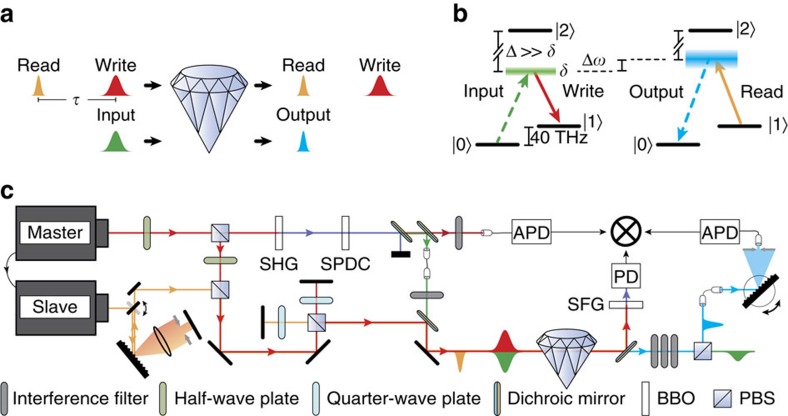
Concept and experiment. (**a**) Input signal photons stored in the diamond by the strong write pulse can be retrieved with modified spectral properties upon output. The output spectrum is controlled by the spectrum of the read pulse. (**b**) Photons are Raman-absorbed to create optical phonons (|1〉), 40 THz above the ground state (|0〉). A read pulse of tunable wavelength and bandwidth retrieves the photon, determining its spectrum. Here, Δ is the detuning from the conduction band (|2〉), *δ* is the input photon bandwidth, Δ*ω* is the detuning between input and output frequencies. (**c**) The master laser (red) is split between the write field and photon source. In the photon source, frequency-doubled laser light pumps SPDC and heralded input signal photons (green) are generated. Signal photons are Raman-absorbed into the optical phonon modes in the diamond via the write field. The slave laser (orange) emits the read field which retrieves the photon from the diamond after time 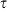. The output signal photon (blue) spectrum is measured on a monochromator. The SFG of read and write pulses triggers the experiment. Coincident detections of output, herald photons and SFG events are measured by a coincidence logic unit. SFG, second-harmonic generation; SPDC, spontaneous parametric downconversion; APD, avalanche photodiode; PD, photodiode; SFG, sum-frequency generation; BBO, β-barium borate; PBS, polarizing beamsplitter.

The frequency converter is based on a quantum memory^6^ modelled by the Λ-level system shown in Fig. 1b, where an input signal photon (723.5 nm centre wavelength and bandwidth *δ*=4.1 nm full-width at half-maximum (FWHM)) and a strong write pulse (800, 5 nm FWHM) are in Raman resonance with the optical phonon band (frequency 40 THz). The large detuning of both fields from the conduction band (detuning Δ≈950 THz) allows for the storage of high-bandwidth photons, while the memory exhibits a quantum-level noise floor even at room temperature^6^. The input signal photon is stored in the memory by Raman absorption with the write pulse, creating an optical phonon. After a delay 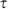, the read pulse annihilates the phonon and creates a modified output photon. By tuning the wavelength and bandwidth of the read pulse, we convert the wavelength of the input signal photon over a range of 17 nm as well as performing bandwidth compression to 2.2 nm and expansion to 7.6 nm (FWHM). The diamond memory is ideally suited to this task, offering low-noise frequency manipulation of THz-bandwidth quantum signals at a range of visible and near-infrared wavelengths in a robust room-temperature device^35^.

The experimental setup is shown in Fig. 1c. The master laser for the experiment is a Ti:sapphire oscillator producing 44 nJ pulses at a repetition rate of 80 MHz and a central wavelength of 800 nm. This beam is split in two parts: the photon-source pump and the write field. In the photon source, the SHG of the laser light pumps collinear type-I spontaneous parametric downconversion (SPDC) in a *β*-barium borate (BBO) crystal, generating photons in pairs, with one at 723.5 nm (input signal) and the other at 894.6 nm (herald). The herald photon is detected on an avalanche photodiode (APD), while the input is spatially and spectrally filtered and overlapped with the orthogonally polarized write pulse on a dichroic mirror. The input signal photon and write field are incident on the 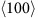 face of the diamond and the input is Raman-absorbed.

The photon is retrieved from the diamond using a read pulse produced by a second Ti:sapphire laser (slave), whose repetition rate is locked to the master, but whose frequency and bandwidth can be independently modified. In this experiment we vary the read field wavelength between 784 and 814 nm, and its bandwidth between 2.1 and 12.1 nm FWHM. To narrow the bandwidth of the read pulse, a folded-grating 4*f*-system^16^ with a narrow slit is used, while in all other configurations the 4*f* line is removed. The read pulse is then overlapped with the write on a polarizing beamsplitter, arriving at the diamond a time 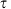 after storage. The horizontally polarized read pulse retrieves a vertically polarized photon (output) from the diamond with spectral shape close to that of the read pulse, blue-shifted by the phonon frequency (40 THz).

The read and write pulses are separated from the signal photons after the diamond by a dichroic mirror; sum-frequency generation (SFG) of the pulses is detected on a fast-photodiode (PD) and used to confirm their successful overlap. Frequency-converted output photons are separated from any unstored input photons by a polarizing beamsplitter, coupled into a single-mode fibre and directed to a monochromator. The spectrally filtered output from the monochromator is coupled into a multi-mode fibre and detected on an APD. Coincident detections between output, herald and read–write SFG events are measured; the experiment is triggered by the joint detection of a herald photon and an SFG signal. (see Methods for further details.)

### Frequency shifts

**Figure 2:**
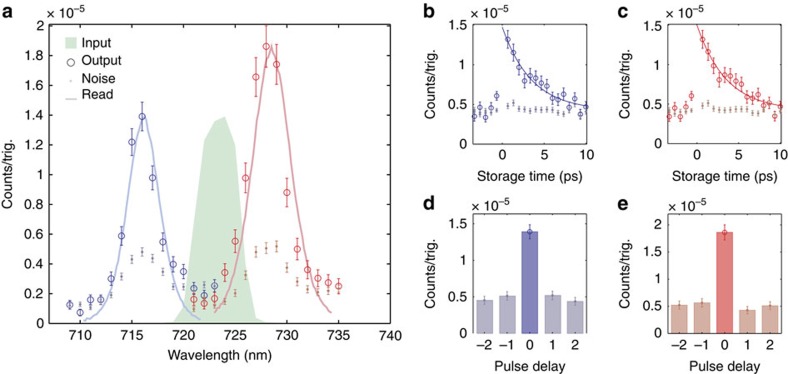
Frequency conversion. (**a**) The measured blue- and red-shifted output photon spectra (hollow circles), and noise (dots), when the read beam is tuned to 792 and 808 nm, respectively. Corresponding read beam spectra, blue-shifted by the phonon frequency, are shown (solid lines) for reference along with the input photon spectrum (green). (**b**) Retrieved blue- and (**c**) Red-shifted signal (hollow circles), and noise (dots), as read–write delay is scanned. An exponential fit gives a phonon lifetime of 3.5 ps. (**d**) Coincidence detection events between blue- and (**e**) red-shifted output and herald photons while scanning the electronic delay between them, as measured at the peak of the spectrum. (**a**–**e**) Error bars show 1 standard deviation calculated assuming Poissonian noise.

Frequency conversion of the signal photon is observed by tuning the slave laser wavelength. We vary this from 784 to 812 nm and measure the output photon wavelength using the monochromator, recording threefold coincidence events. The resulting output spectra, with the read pulse centred at 792 and 808 nm, are shown in Fig. 2a (hollow circles). The spectrum of each read pulse (solid lines), blue-shifted by the phonon frequency, is plotted alongside the relevant output photon spectrum to show how the photon spectrum is determined by the read pulse. We find the peaks of the output spectra to be 716 and 728 nm with bandwidths 3.3 and 3.5 nm, FWHM respectively, making the output spectrally distinguishable from the input (green).

Following retrieval, the time-correlations characteristic of SPDC photon-pairs are preserved. This is measured by scanning the electronic delay between the signal and herald detection events in steps of 12.5 ns (the time between adjacent oscillator pulses) and counting coincident detections. Results are shown in Fig. 2d,e for blue- and red-shifted cases, respectively. We quantify this using the two-mode intensity cross-correlation function between output signal and herald fields given by 
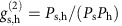
. Here, *P*_s_(*P*_h_) is the probability of detecting a photon in the signal (herald) mode, and *P*_s,h_ is the probability of measuring a joint detection. A measurement of 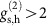 indicates non-classical correlation^36^,^37^ (see Methods), whereas uncorrelated photon detections, for example, from noise, give 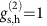. We calculate the values of 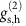 at the peak of the blue- and red-shifted spectra to be 2.7±0.2 and 3.4±0.3, respectively.

Figure 2b,c shows the blue-(red-)shifted photon retrieval rate as a function of the optical delay 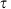 between read and write pulses. An exponential function is fit to the data and we find a memory lifetime of 3.5 ps as expected from the lifetime of the optical phonon^35^,^38^. This exceeds 12 times the duration of the input photon (see Methods). Also plotted in Fig. 2a–c are the measured coincidences due to noise (dots), which are measured by taking the average of the ±12.5 and ±25 ns time-bins as shown in Fig. 2d,e. Noise comes from two processes: four-wave mixing^35^; and read pulses scattering from thermally populated phonons producing anti-Stokes light^6^,^35^. The latter can be reduced by an order of magnitude by cooling the diamond to −60 °C.

**Figure 3:**
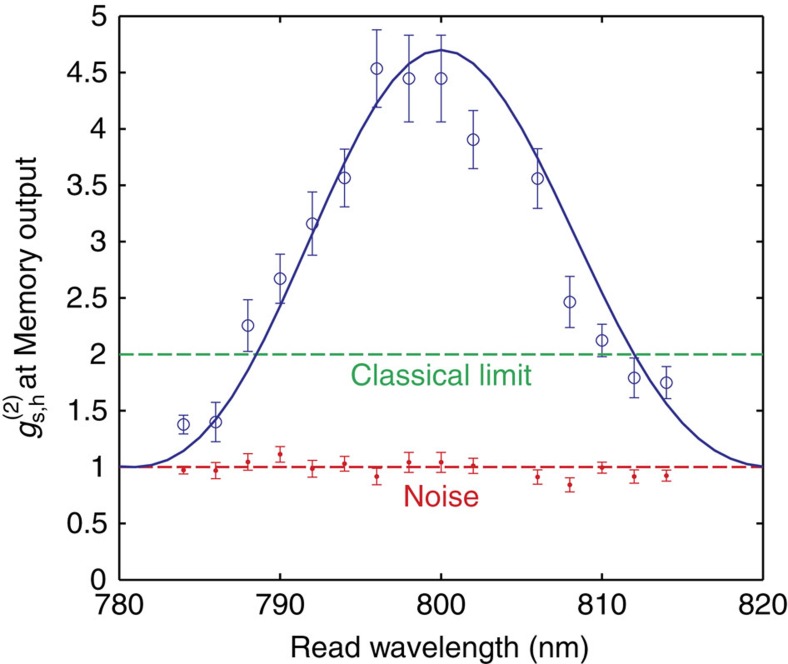
Range of frequency conversion. Measured 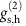 of frequency-shifted photons. Frequency conversion, tuning the read beam over a 30 nm range, is observed. Non-classical statistics, that is, 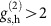, are maintained over a 18 nm range. The estimated 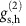 (solid line) which depends on sinc^2^(Δ*kL*/2) agrees well with experimental data suggesting that the range of frequency conversion is determined by phase-matching conditions. Error bars show 1 standard deviation calculated assuming Poissonian noise.

Figure 3 shows 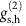 measured at the peak of each output signal spectrum as the read wavelength is tuned over a 30 nm range. We find that blue- and red-shifted single photons maintain non-classical correlations over a 17 nm range. Since noise is uncorrelated with herald photons, we expect the noise to have cross-correlation *g*^(2)^=1. The 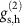 will increase from 1 in proportion to the signal-to-noise ratio (see Methods for further details),


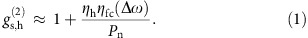


Here *η*_h_=*P*_s,h_/*P*_h_=0.13% is the photon heralding efficiency in the signal arm including the monochromator, *P*_n_=3.8 × 10^−6^ is the probability of detecting a noise photon, and *η*_fc_(Δ*ω*) is the conversion efficiency as a function of frequency detuning, Δ*ω*, between input and output photons. The conversion efficiency *η*_fc_(Δ*ω*)=*η*_fc_(0) × sinc^2^(Δ**k***L*/2), where *L*=2.3 mm is the length of the diamond along the propagation axis, and Δ**k**=**k**_i_−**k**_o_+**k**_r_−**k**_w_ is the phase mismatch between the input signal (i), output signal (o), read (r) and write (w) fields because of material dispersion in diamond^35^. The conversion efficiency was measured to be *η*_fc_(0)=1.1%, inside the diamond at zero detuning. As the diamond is not anti-reflection coated, a 17% reflection loss occurs at each face.

Inserting experimental parameters into equation 1 returns 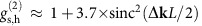, which is plotted along side data in Fig. 3 (solid line). The close agreement with experiment suggests that the limitation on frequency conversion comes primarily from phase-matching conditions. We then expect that the range of frequency conversion in diamond can be extended by modifying the phase-matching conditions. This could be achieved by shortening the diamond crystal, or by employing non-collinear beam geometries^39^. In the current configuration, the maximum conversion efficiency is limited to ∼1% due to the efficiency of the quantum memory^6^. However, we note that this could be improved with increased intensity in read and write pulses, or by increasing the Raman coupling, for example, by the use of a waveguide.

### Bandwidth manipulation

**Figure 4:**
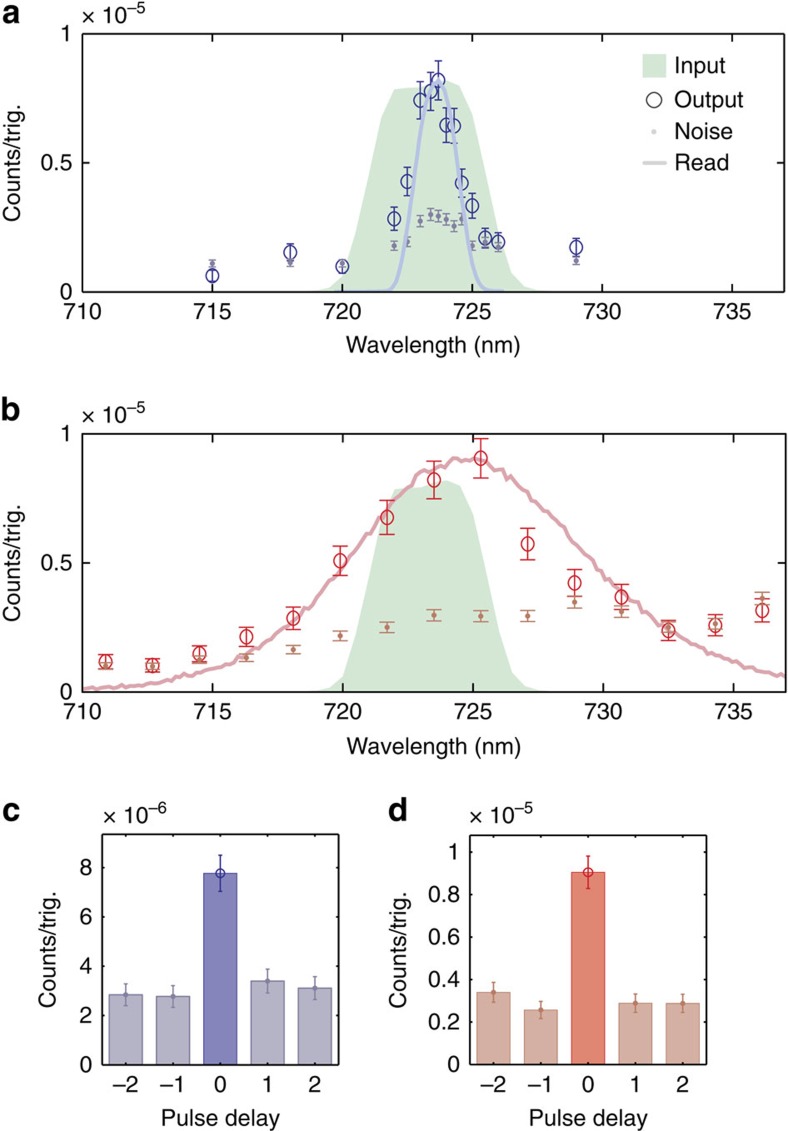
Bandwidth conversion. (**a**) Narrowed output spectrum (hollow circles) and noise (dots) with read beam FWHM at 2.1 nm. (**b**) Expanded output photon spectrum (hollow circles) and noise (dots) with read beam FWHM at 12.1 nm. (**a**,**b**) Corresponding read beam spectra, blue-shifted by the phonon frequency, are shown (solid lines) for reference along with the input photon spectrum (green). (**c**) Coincidence detection events between bandwidth narrowed output photons, and (**d**) expanded output photons and heralds while scanning the electronic delay between them, as measured at the peak of the spectrum. (**a**–**d**) Error bars show 1 standard deviation calculated assuming Poissonian noise.

Bandwidth conversion is observed by tuning the slave laser bandwidth. With the read pulse wavelength centred at 801 nm, its bandwidth could be tuned from 12.1 to 2.1 nm FWHM using a slit in a grating 4*f* line. Figure 4a,b shows the resulting narrowed (expanded) output photon spectrum with the corresponding read pulse spectrum, blue-shifted by the phonon frequency, and the input signal photon spectrum for reference. The resulting narrowed and expanded photon bandwidths are 2.2 and 7.6 nm, FWHM, respectively. Figure 4c,d shows the conservation of timing correlations between bandwidth-narrowed (-expanded) photons and herald photons, respectively. We measure 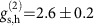 in the narrowed bandwidth case, 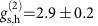 in the expanded bandwidth case, showing that bandwidth-converted output light from the diamond maintains non-classical correlations with herald photons.

## Discussion

We have demonstrated ultrafast quantum frequency manipulation by adjusting the central wavelength and spectrum, of THz-bandwidth heralded single photons. We achieve this spectral control using a modified Raman quantum memory in diamond. The single photons are written to the memory from one spectral mode, and recalled to another. Critically—and unlike frequency conversion based on, for example, amplification—the non-classical photon statistics in our demonstration were retained after spectral manipulation. Diamond therefore offers low-noise THz-bandwidth storage and frequency control of single photons on a single, robust, room-temperature platform. We have demonstrated frequency conversion of a single polarization; two memories could be used in parallel to convert a polarization qubit. Quantum memories for long-distance quantum communication typically demand long storage times at the expense of high bandwidth; this application leverages a high-bandwidth memory where long storage time is not relevant. We believe that this system could find use in a number of applications, including entangling two photon-pair sources of different colour; reducing the bandwidth of the ubiquitous ultrafast SPDC photon source, without losing photons (as occurs in passive filtering); broadening the bandwidth of an SPDC photon source to match a chosen material system (not possible with a passive filtering system); and increasing the dimensionality of quantum encoded information^15^. Ultimately, we believe that arbitrary optical function generation^40^—with both classical and quantum light—and associated signal processing capabilities will be a platform for future technology development. We expect that the large-bandwidth nonlinear optical conversion of Raman-based quantum memories will find use in implementations of these generators.

## Methods

### Photon source

Laser light is frequency-doubled by type-I SHG in a 1 mm BBO crystal before pumping collinear type-I SPDC in a second 1 mm BBO crystal. Horizontally polarized photon pairs are generated at 894.6 and 723.5 nm. Remaining pump light is filtered out and photon pairs are separated by a 801 nm long-pass dichroic mirror. The 894.6 nm photon passes through a 5 nm interference filter, is coupled into a single-mode fibre, and detected on an APD. A detection heralds the presence of the 723.5 nm photon, which is spatially filtered in a single-mode fibre and spectrally filtered by an interference filter with bandwidth of 5 nm (FWHM). The input and write pulses are overlapped using a 750 nm shortpass dichroic mirror. The input and write pulses are focused into the diamond by an achromatic lens of focal length 6 cm.

### Diamond

The diamond is a high-purity, low birefringence crystal grown by chemical vapour deposition by Element Six Ltd. The crystal is 2.3 mm long, cut along the 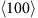 lattice direction, and polished on two sides.

### Storage time

Absorption of the input photon by the diamond lattice is observed by an 18% dip in input-herald coincidences when the input photon and write field arrive at the diamond simultaneously. The duration of the input photon can be deconvolved from the width of the absorption dip, 346 fs. With write pulses 190 fs in duration, the input photon pulse duration time is 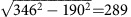 fs, assuming transform-limited Gaussian pulses. The characteristic storage time of the diamond memory is 3.5 ps, found from an exponential fit to storage data, over 12 times the duration of the input pulse.

### Laser locking

The repetition rate of the slave laser is locked to that of the master using a Spectra Physics Lok-to-Clock device. We send read and write beams through a cross-correlator (type-II SFG in a 1 mm BBO crystal) and detect the resulting signal on a PD confirming that the time difference between the two pulses is ≤200 fs. We measure a typical SFG signal rate of 2.5 MHz; we use this signal to trigger the experiment.

### Monochromator

The monochromator (Acton SP2300) is comprised of a 1,200 g mm^−1^ grating between two 30 cm focal length spherical mirrors. The output is coupled to a multi-mode fibre (105 μm core). The apparatus has a spectral resolution of 1.1 nm and an overall efficiency of 10% at 723 nm.

### Cross-correlation function

The cross-correlation function between the herald and frequency-converted light is given by 
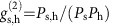
. Classically, 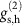 is upper-bounded by a Cauchy–Schwarz inequality^36^,^37^ 
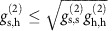
. Here, the terms on the right-hand side are the intensity auto-correlation functions for the output signal and herald fields, which we assume, being produced by SPDC, follow thermal statistics and have 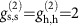. Adding any uncorrelated noise would strictly lower terms on the right-hand side towards 1. To model the effect of noise on this measurement, we assume that the signal is made up of a mixture of noise photons (detected with probability *P*_n_=3.8 × 10^−6^) and frequency-converted photons (probability *P*_*γ*_), such that


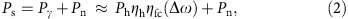



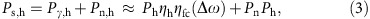


where *η*_h_=1.3 × 10^−3^ is the heralding efficiency which equates to the collection efficiency of the entire signal arm, including the monochromator, and *η*_fc_(Δ*ω*) is the efficiency of the quantum frequency conversion. This returns


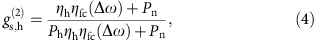


from which equation 1 follows, given that 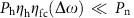.

### Background subtraction

When measured at the photon source the input and herald photon cross-correlation is 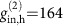. Because of imperfect polarization extinction the input photon can, with low probability, traverse the monochromator and be detected, thereby artificially inflating the measured 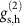 of the converted output. For this reason we make a measurement with no read/write pulses present and subtract these counts from the output signal when read/write pulses are present to portray an accurate value of 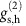. As an example, in Fig. 3, the peak count rate is 19 × 10^−6^ photons per pulse compared with a background of 3 × 10^−6^ photons per pulse.

## Additional information

**How to cite this article:** Fisher, K. A. G. *et al.* Frequency and bandwidth conversion of single photons in a room-temperature diamond quantum memory. *Nat. Commun.* 7:11200 doi: 10.1038/ncomms11200 (2016).
